# Recent advances in CGG repeat diseases and a proposal of fragile X-associated tremor/ataxia syndrome, neuronal intranuclear inclusion disease, and oculophryngodistal myopathy (FNOP) spectrum disorder

**DOI:** 10.1038/s10038-022-01116-y

**Published:** 2023-01-20

**Authors:** Hiroyuki Ishiura, Shoji Tsuji, Tatsushi Toda

**Affiliations:** 1 Department of Neurology, Graduate School of Medicine, The University of Tokyo, Tokyo, Japan; 2 Department of Neurology, Okayama University Graduate School of Medicine, Dentistry and Pharmaceutical Sciences, Okayama, Japan; 3 Institute of Medical Genomics, International University of Health and Welfare, Narita, Japan

**Keywords:** Genetics research, Disease genetics

## Abstract

While whole genome sequencing and long-read sequencing have become widely available, more and more focuses are on noncoding expanded repeats. Indeed, more than half of noncoding repeat expansions related to diseases have been identified in the five years. An exciting aspect of the progress in this field is an identification of a phenomenon called repeat motif–phenotype correlation. Repeat motif–phenotype correlation in noncoding repeat expansion diseases is first found in benign adult familial myoclonus epilepsy. The concept is extended in the research of CGG repeat expansion diseases. In this review, we focus on newly identified CGG repeat expansion diseases, update the concept of repeat motif–phenotype correlation in CGG repeat expansion diseases, and propose a clinical concept of FNOP (fragile X-associated tremor/ataxia syndrome, neuronal intranuclear inclusion disease, and oculopharyngodistal myopathy)-spectrum disorder, which shares clinical features and thus probably share some common disease pathophysiology, to further facilitate discussion and progress in this field.

## Introduction

Recently, increasing attention has been paid to noncoding repeat expansion diseases; indeed, more than half of noncoding repeat expansion diseases have been identified after 2017. Of course, whole genome sequencing played an essential role in the identification of these repeat expansions, and nowadays long-read sequencing technology including single-molecule real-time sequencing and nanopore sequencing is a powerful tool to detect repeat expansions. For example, genetic causes of benign adult familial myoclonus epilepsy (BAFME) have long been explored, which is characterized by autosomal dominant inheritance, adulthood-onset cortical myoclonus, and infrequent epilepsy with a benign course as compared to progressive myoclonic epilepsy. Whereas this disease is also called familial adult myoclonic epilepsy (FAME) or familial cortical myoclonic tremor with epilepsy (FCMTE), we believe we should keep this wording since the disease was first described and the clinical entity was well established by many Japanese researchers under the name of BAFME. In 2018, we identified TTTCA and TTTTA repeat expansions in an intron of *SAMD12* as the cause of BAFME type 1 (BAFME1) [1]. While expansions of TTTTA repeats were found in a limited number of controls (5.9%), expanded TTTCA repeats were not found in controls, which strongly suggested TTTCA, rather than TTTTA, has an important role in the pathogenesis of BAFME. From the remaining two families without repeat expansions in *SAMD12*, we identified expansions of the same repeat motifs in introns of *TNRC6A* and *RAPGEF2* and named the disease BAFME6 and BAFME7, respectively. Again, expansions of TTTTA repeats were observed in 0.5% and 0.2% of controls but no TTTCA repeats were found in controls in *TNRC6A* and *RAPGEF2*. From the study, we postulated a novel concept, “repeat motif–phenotype correlation,” in the noncoding repeat expansion diseases [2]. The concept was further supported by the identification of expanded TTTCA and TTTTA repeats in *STARD7*, *MARCHF6*, and *YEATS2* in BAFME types 2, 3, and 4, respectively [3–5]. We found an aggregation of RNA molecules (RNA foci) consisting of UUUCA repeats in neurons of the autopsied brains of patients with BAFME1. From the observation, RNA-mediated gain-of-function pathomechanism is postulated [1].

On the basis of the findings, we moved on to the other neurological and muscular diseases, namely, neuronal intranuclear inclusion disease (NIID), oculopharyngeal myopathy with leukoencephalopathy (OPML), and oculopharyngodistal myopathy (OPDM). We noted some overlap in clinical features in these diseases and fragile X-associated tremor/ataxia syndrome (FXTAS) caused by CGG repeat expansions in *FMR1* [6]. We searched for CGG repeat expansions and finally concluded that these diseases are caused by CGG repeat expansions in *NOTCH2NLC*, *LOC642361*/*NUTM2B-AS1*, and *LRP12* [7]. Thereafter, CGG repeat expansions in *GIPC1* [8, 9] and CCG repeat expansions in *RILPL1* [10] are also found to cause oculopharyngodistal myopathies. Very interestingly, patients who have CGG repeat expansions in *NOTCH2NLC* can also show oculopharyngodistal myopathy phenotype called OPDM3 [11, 12]. In other words, these lines of evidence indicate that CGG or CCG repeat expansions can cause a novel clinical spectrum from leukoencephalopathy to oculopharyngeal type myopathy. This review focuses on the clinical spectrum of the disorders caused by CGG repeat expansions.

## FXTAS

### A brief history of FXTAS

Fragile X syndrome (FXS) is an X-linked disorder characterized by developmental delay and intellectual disability. A fragile site FRAXA was originally identified in patients with FXS by cytogenetic analysis with a folate-deficient media. Thereafter, FXS is revealed to be caused by CGG repeat expansions in the 5ʹ untranslated region (UTR) of *FMR1*, encoding FMRP. Lengths of CGG repeat units in FXS are usually >200, which are called full mutations. The full mutations cause hypermethylation of CpG sites, leading to transcriptional silencing of *FMR1*. In line with that, deletions, nonsense, or frameshift mutations in *FMR1* are also found in patients with FXS [13].

FXTAS was initially recognized by the finding that some of the family members of patients with FXS present late-onset parkinsonism, tremor, and cognitive decline [6]. These patients were found to have shorter CGG repeats in *FMR1*, namely, 55–200 repeat units called premutation. It has been shown that the expression level of *FMR1* is not silenced but rather increased (several times) in FXTAS. Now accumulation of abnormal RNA, including expanded CGG repeats or subsequent abnormal biological processes such as repeat-associated non-AUG-initiated translation (RAN translation), is considered the pathomechanism. Thus, many consider there is a distinct pathomechanism in FXTAS such as gain-of-toxic function compared to FXS where expression of *FMR1* is silenced [14].

### Clinical features of FXTAS

Penetrance of FXTAS in male carriers aged 50 years is about 40%, whereas that of female carriers is 16% [15, 16]. The age at onset is typically about 60 years of age. Neurological signs include intention tremor, cerebellar ataxia, cognitive decline including frontal executive dysfunction, peripheral neuropathy, and dysautonomia. Some show mild Parkinsonism and psychiatric manifestations. Characteristic neuroradiological findings are white matter lesions (T2/FLAIR hyperintensity) in middle cerebellar peduncles (MCP sign), white matter lesions (T2/FLAIR hyperintensity) in the cerebrum and the splenium of the corpus callosum, and cerebral and cerebellar atrophy. Although frequencies seem low, some patients with FXTAS show diffusion-weighted MRI hyperintensities in the corticomedullary junctions, similar to those of NIID [7, 17]. Carrier females generally have less severe manifestations than males. Premature ovarian failure can be associated with female carriers, and the condition is called fragile X-associated premature ovarian failure (FXPOI).

While this condition is common in Western countries (1/813 males have premutation alleles in Canada) [18], the frequencies of FXTAS, and thus FXS, is relatively uncommon in Japan. For example, FXTAS is found in only 0.3–1.1% of undiagnosed cerebellar ataxia in Japan [19, 20].

## NIID

### Molecular genetics of NIID1 (*NOTCH2NLC*-related disorder)

The concept “neuronal intranuclear inclusion disease” was originally described in a case of an autopsied patient [21]. The disease is recognized only by autopsy and rarely reported until recently [22–25].

After Sone et al. reported the usefulness of skin biopsy for antemortem diagnosis of NIID [26, 27] and the diagnostic usefulness of diffusion-weighted MRI hyperintensities in the corticomedullary junctions have widely been established, many cases started to be diagnosed. In 2019, CGG (or GGC) repeat expansions in *NOTCH2NLC* (formerly annotated as *NBPF19*) were identified in patients with NIID [7, 28, 29].

CGG repeats in *NOTCH2NLC* are widely observed in patients with NIID in East Asia, including Japan [7, 30], China [29], Malaysia [7, 30], Taiwan [31, 32], and Singapore [33]. At least in Japan, most patients with clinically typical presentations have expanded CGG repeats in *NOTCH2NLC*, including patients without an obvious family history. On the contrary, CGG repeat expansion in *NOTCH2NLC* is rarely seen in Western countries. In particular, expanded CGG repeats in patients reported having NIID were excluded [34] in previously reported cases [22–24]. Therefore, to avoid confusion between pathological and genetic definitions, we should better use the term NIID type 1 [7] or *NOTCH2NLC*-related disorder for patients with expanded CGG repeats in *NOTCH2NLC*.

Sporadic cases of NIID1 are frequently observed, suggesting that the penetrance of CGG repeat expansions in *NOTCH2NLC* is incomplete. Incomplete penetrance may be due to the late onset or underrecognition of the disease in the former generations. A recent study using genomic DNA extracted from blood or lymphoblastoid cell lines [35] suggested very long CGG repeats in *NOTCH2NLC* in unaffected fathers in four families cause methylation and transcriptional silencing, presumably sparing the gain-of-toxic effect of the expanded repeats. The mechanism can also contribute to the low penetrance of the disease.

### Clinical features of *NOTCH2NLC*-related disorders

The age at onset of the majority of patients with NIID1 is after 50 years, while a limited number of patients show childhood- or juvenile-onset. Clinical features include cognitive decline including frontal executive dysfunction, peripheral neuropathy, autonomic dysfunction, tremor, and cerebellar ataxia [36]. Although less noted by patients, detailed ophthalmological examination frequently reveals retinopathy in patients with NIID1 (*NOTCH2NLC*-related retinopathy) [37, 38].

Sone et al. classified the patients into “dementia-dominant group” and “limb weakness group” by the initial manifestations. In the dementia-dominant group, most patients show cognitive decline and miosis. About half of patients show cerebellar ataxia and bladder dysfunction. Less frequent clinical signs include tremor, rigidity, and abnormal behavior. Note that some patients show vomiting, disturbance of consciousness, or encephalitis-like episodes, all of which can occur recurrently.

Leukoencephalopathy and diffusion hyperintensity in the corticomedullary junction are characteristic findings in brain MRI. MCP signs are also observed [7], and paravermal hyperintensity is another neuroradiological finding in NIID1 [39, 40], all of which are found in FXTAS [41]. Reflecting peripheral neuropathy, slowing of motor and sensory nerve velocities with or without decreased amplitudes of compound muscle action potentials or sensory nerve action potentials are also frequently observed. Biopsied skin, as well as autopsied tissues, showed eosinophilic ubiquitin- and p62-positive intranuclear inclusions. Electron microscopy reveals filamentous inclusions composed of filaments [26, 27] with a diameter of 6–9 nm [42].

## OPDM and related disorders

### Molecular genetics of OPDM and related diseases

OPDM was originally reported by Satoyoshi and Kinoshita [43] from Japan, where they presented four families with OPDM. The mode of inheritance was considered to be autosomal dominant. Muscle biopsy revealed myopathic changes with rimmed vacuoles. After the discovery of exonic GCG/GCA repeats in *PABPN1* encoding polyalanine stretch in oculopharyngeal muscular dystrophy (OPMD), OPDM was clinically and genetically recognized as a distinct disease from OPMD [44, 45]. Distal predominant weakness and facial weakness are generally more common in OPDM than OPMD, whereas proximal predominant weakness is more common in OPMD. Pathologically, intranuclear inclusions of a diameter of 8.5 nm are specific for OPMD [44]. Most cases of OPDM were reported from Japan and China, whereas only 13 families were reported from other regions such as Thai [46], Netherlands [47], Turkey [48], England [49], and Italy [50] until 2019.

We identified a family presenting oculopharyngeal type myopathy. The mode of inheritance is autosomal dominant. The proband is complicated by tremor, leukoencephalopathy, and intestinal pseudo-obstruction in addition to dilated cardiomyopathy and respiratory failure. We named the disease oculopharyngeal myopathy with leukoencephalopathy (OPML). Of note, diffusion hyperintensity is found in the corticomedullary junction. By hypothesizing that CGG repeat expansions also cause OPML because MRI features are similar to those observed in NIID1, we identified CGG repeat expansions in chromosome 10 in the family (named OPML1). There are bidirectionally transcribed noncoding genes, namely, *LOC642361* (CGG direction) and *NUTM2B-AS1* (CCG direction) [7]. The expanded repeats are cosegregated in the family (four affecteds and seven unaffecteds) and not found in 1,000 controls.

We then performed a whole genome sequencing analysis of a patient with OPDM because distributions of affected muscles are similar to those of OPML1. The analysis identified CGG repeat expansions in the 5ʹ UTR of *LRP12* as a cause of OPDM (named OPDM1). OPDM1 is shown to be the most common in Japan [51].

Following the study, CGG repeat expansions in the 5ʹ UTR of *GIPC1* were revealed to be the cause of OPDM2 [8, 9]. OPDM2 is most frequent in China, whereas OPDM2 is less frequent in Japan. Recently, CCG repeat expansions in *RILPL1* were also revealed in undiagnosed OPDM (named OPDM4) [12]. Thus, for now, OPDM is strongly related to noncoding CGG or CCG repeat expansions. Some patients, however, are still waiting for molecular diagnosis; further genetic heterogeneity is indicated.

### Clinical features of OPDM

OPDM is characterized by facial, ocular, pharyngeal, and distal muscle weakness. Although distal predominant muscle weakness is a cardinal feature of the disease, muscle weakness is sometimes proximal predominant and asymmetrical. Onset with limb muscle weakness is typical in OPDM1 and OPDM2, whereas many patients with OPDM4 present onset with ptosis or dysphagia [10].

Muscle biopsy reveals myopathic changes with rimmed vacuoles, but rimmed vacuoles are not found in less affected muscles with preserved muscle strengths. Ubiquitin- or p62-positive inclusion is observed. Electron microscopy reveals intranuclear [45, 51] or cytoplasmic [44, 51] inclusions composed of filaments with a diameter of 10–18 nm, which is different from those observed in OPMD composed of filaments with a diameter of 8.5 nm. A case report found intranuclear inclusions in other organs including central nervous system [52]. Intranuclear inclusions were identified by skin biopsy in all six patients with OPDM2 [53]. However, only a part of patients with OPDM1 (1/3) showed intranuclear inclusions in skin biopsy samples [53].

## Proposal of FXTAS, NIID, and oculopharyngodistal myopathy (FNOP) spectrum disorder

**Fig. 1:**
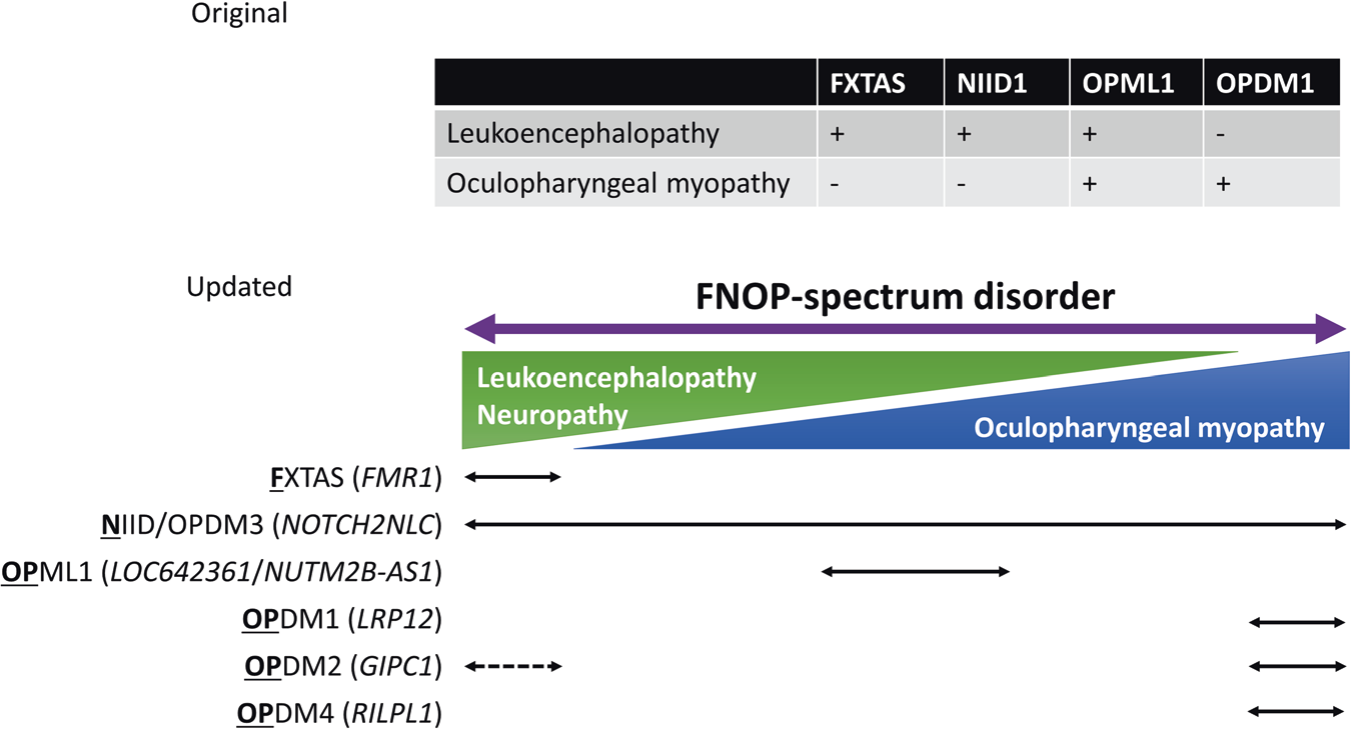
Broadening a concept of FNOP spectrum disorder. The figure shows the original [7] and updated concepts of diseases including fragile X-associated tremor/ataxia syndrome (FXTAS), NIID1 (neuronal intranuclear inclusion disease type 1), OPML1 (oculopharyngeal myopathy with leukoencephalopathy type 1), oculopharyngodistal myopathies (OPDMs), which are caused by expanded CGG repeats. Since the original proposal (upper panel), the clinical spectrum of *NOTCH2NLC*-related disease is broader than previously thought and new types of OPDMs (types 2 and 4) have been identified. Thus, expanded CGG or CCG repeats cause a spectrum of diseases from leukoencephalopathy to oculopharyngodistal myopathy, called an FNOP-spectrum disorder

Our previous study revealed NIID1 (showing leukoencephalopathy and neuropathy), OPML1 (showing both leukoencephalopathy and oculopharyngeal myopathy), and OPDM1 (showing oculopharyngeal myopathy) are caused by CGG or CCG repeat expansions [7]. Taking FXTAS into consideration, we proposed that the findings broadened the concept of repeat motif–phenotype correlation; leukoencephalopathy and oculopharyngeal myopathy form a disease spectrum and expanded CGG or CCG repeats underlie the disease spectrum (Fig. 1).

**Fig. 2:**
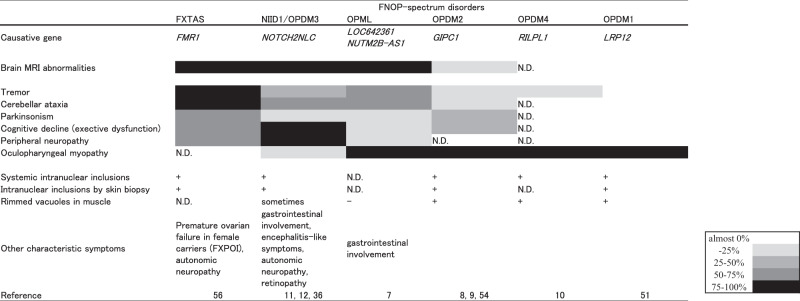
Clinical features of FNOP spectrum disorders. The figure summarizes clinical features of FNOP (fragile X tremor/ataxia syndrome, neuronal intranuclear inclusion disease, and oculopharyngodistal myopathy)-spectrum disorder. ND not described

After our publication, situations became more complicated. Two studies revealed CGG repeat expansions in *NOTCH2NLC* also cause OPDM phenotype (named OPDM3) [11, 12]; about half of patients with OPDM phenotype with the *NOTCH2NLC* expansions (OPDM3) show leukoencephalopathy, neuropathy, or other signs compatible with NIID1. In addition, a recent study found that CGG repeat expansions in *GIPC1* are also observed in patients with movement disorders [54], some of whom showed mild leukoencephalopathy or intranuclear inclusions in skin, although there remain limitations such as unavailability of autopsy as the authors discussed. These findings, however, suggested that the borders between leukoencephalopathy and oculopharyngeal myopathy might be more ambiguous (Fig. 1, Fig. 2).

One problem is that OPDM and NIID need to be better defined. These terms sometimes describe a clinicopathologic diagnosis and may only describe the primary symptoms. Taking *NOTCH2NLC*-related disease as an example, when leukoencephalopathy or neuropathy predominate, the disease is often called NIID. When oculopharyngeal-type myopathy predominates, it is often called OPDM. However, there are cases in which features of both are combined, and extra-muscular symptoms may not be given much attention in patients once given a clinical diagnosis of OPDM. Thus, given the discovery of the molecular basis, the clinical presentations have become much broader than previously thought.

In addition to FXTAS, which is originally described, NIID1/OPDM3, OPML1, OPDM1, OPDM2, and OPDM4 shares some similarities as listed below. (1) They are caused by expansions of noncoding CGG or CCG repeats. (2) Most cases are late- or adulthood-onset. (3) Core features include leukoencephalopathy, neuropathy, and oculopharyngeal-type myopathy. MRI findings share similar findings such as MCP signs [7], paravermal hyperintensities [39–41], or diffusion hyperintensities in the corticomedullary junction [7, 17, 41] in addition to white matter changes in the cerebrum. However, there is substantial interfamilial and intrafamilial variability in clinical status and neuroradiological findings [40]. (4) Ubiquitin- or p62-positive inclusions are a pathological hallmark. 5. Penetrance seems incomplete; these diseases have many sporadic cases.

These lines of common clinical features, in addition to the same repeat motif (CGG), which is expanded in the patients, strongly indicate that there are common pathways in these diseases, probably caused by the gain-of-function effect of expanded CGG repeats. Each disease name, however, is named individually and historically, and now we believe a new term indicating the novel clinical spectrum is needed. Here, we propose the name “FXTAS, NIID, and oculopharyngeal myopathy (FNOP)-spectrum disorder” in these names to facilitate further clinical discussion and genetic investigation. More specifically, we should examine in detail the symptoms and clinicopathological findings that can occur in FNOP-spectrum disorder in all patients. Then, we should examine the frequency of each symptom and clinicopathological findings associated with each expanded repeat, which would provide more convincing clinical presentations based on molecular diagnosis than giving a clinical diagnosis of NIID or OPDM based on predominant clinical presentations. This concept is also useful when discussing cases with undetermined causes.　For example, although detailed molecular analysis has not been reported, a patient reported by Amato et al. [55] shows oculopharyngodistal myopathy with intestinal pseudo-obstruction can fall into this category because vomiting and intestinal pseudo-obstruction probably due to autonomic neuropathy can also be seen in patients with NIID1 and OPML1. It should be noted that other diseases, such as mitochondrial disease, can mimic the disease spectrum, although mitochondrial DNA analysis and assays for mitochondrial enzyme activity were revealed to be normal in the patient [55].

## Conclusions and future perspectives

In this review, we summarized recent advances in CGG repeat disorders and proposed a concept of FNOP spectrum disorder mainly from the clinical and genetic aspects of view. The main questions are as follows. How many other genes are involved in FNOP spectrum disorders? What are the key common pathomechanisms? How are affected sites determined? In other words, why do some patients show leukoencephalopathy, but others show oculopharyngodistal myopathy? What is the effective therapeutic approach? Finally, we have to proceed to understand these diseases further.
